# The Microvasculature After Reperfused Myocardial Infarction: To Examine or Not to Examine?

**DOI:** 10.1161/JAHA.112.003392

**Published:** 2012-08-24

**Authors:** Christopher M. Kramer

**Affiliations:** From the Departments of Medicine and Radiology and the Cardiovascular Imaging Center, University of Virginia Health SystemCharlottesville, VA

**Keywords:** editorials, microcirculation, primary percutaneous coronary intervention, magnetic resonance imaging, myocardial infarction, ventricular remodeling

fter acute reperfused myocardial infarction (AMI), there is a variable degree of microvascular damage due to reperfusion injury. This injury is multifactorial, and potential culprits include cellular inflammation (leukocytes and macrophages), reactive oxygen species, shifts in metabolic substrate, and alterations in mitochondrial and cytosolic calcium ion homeostasis.^1^ Characterization of reperfusion injury has evolved steadily over the past 2 decades. Imaging has played a leading role, including such techniques as contrast echocardiography,^2^ x‐ray angiography with measurement of myocardial perfusion grade,^3^ and contrast‐enhanced cardiac magnetic resonance (CMR) imaging.^4,5^

Markers of reperfusion injury on CMR include microvascular obstruction, which appears as regions of hypointense signal in the first few minutes after gadolinium contrast administration. CMR studies have demonstrated that microvascular obstruction is associated with poor outcome for the particular segment of myocardium in which it occurs,^6^ for the left ventricle (LV) over time because of adverse remodeling,^7^ and for the patient in terms of risk of morbidity and mortality.^5,8^ In addition, myocardial hemorrhage has been recognized as another marker of severe injury^9^ and can be imaged on T2‐weighted MRI as areas of low signal intensity. Yet another marker of prognosis by CMR is myocardial salvage index. Myocardial salvage index is calculated as the difference between the area at risk, identified by T2‐weighted (W) imaging as regions of bright signal consistent with myocardial edema,^10^ and infarct size, which is measured by late gadolinium enhancement.^11^ In a recent study of patients followed up for 6 months after AMI, myocardial salvage index was a powerful predictor of outcome, inasmuch as nearly all of the cardiac events occurred in patients with myocardial salvage index less than the median.^12^

In the past few years, a catheter‐based technique has been developed for assessment of microvascular injury in the setting of AMI—namely, the index of microcirculatory resistance (IMR).^13^ IMR is measured with a pressure sensor / thermistor–tipped guidewire and is calculated as mean distal coronary pressure × mean transit time of a 3‐cc saline bolus measured during adenosine hyperemia. Fearon et al^13^ demonstrated in 29 patients after primary percutaneous coronary intervention for AMI that IMR correlated significantly with 3‐month post–myocardial infarction echocardiographic wall motion score and was its strongest predictor on multivariate analysis. IMR also predicted recovery of regional LV function.

In this issue of the *Journal of the American Heart Association (JAHA),* Payne et al^14^ studied the use of this technique in a larger cohort (n=108) of post‐AMI patients and compared the results with CMR studies at 2 days after AMI and 3 months after AMI. The authors are to be congratulated on the completion of this tour‐de‐force, which involved a large number of patients and 2 follow‐up imaging studies, for which they had an 89% completion rate. They found that the measurement of IMR was extremely reproducible. IMR was a multivariate predictor of early myocardial salvage and predicted the presence and extent of microvascular obstruction and myocardial hemorrhage. In anterior myocardial infarction, IMR predicted late myocardial salvage. Thus, a catheter‐based measurement in AMI can accurately assess the extent of microvascular injury while percutaneous coronary intervention is being performed, which is a potential way to obviate the need for further imaging of microvascular injury. Still, the correlations with 3‐month ejection fraction and end‐systolic volumes were modest and, in fact, fell out in multivariate models because IMR correlated much better with baseline measures of acute microvascular injury on CMR. This could have been, at least in part, because the infarct sizes were modest and ejection fractions fairly preserved, and thus the subsequent LV remodeling, which is dependent on initial infarct size, was likewise modest.

Several questions are raised by this interesting study. First, does the IMR measure add to what we already know at the time of intervention? The answer is a decided *yes,* given that measurement of Thrombolysis In Myocardial Infarction (TIMI) grade is an inadequate way of assessing microvascular function. Next, does IMR obviate performance of further imaging, such as CMR to assess the status of the microvasculature? This is a more difficult question to answer. Certainly, IMR adds to the understanding of myocardial physiology after reperfused myocardial infarction. One could ask, even though IMR correlated with the presence and extent of microvascular obstruction and myocardial hemorrhage, “Why not just perform the CMR and get a complete and definitive evaluation?” Certainly, cost and availability are issues with CMR. Not every hospital has CMR capability, and the additive cost of a study is not inconsequential. Payne et al^14^ contend in the introduction to their article that CMR is “usually performed up to 7 days after hospital admission in medically stabilized patients.” Perhaps that is true in Scotland. In the United States, hospital stays are generally on the order of 2 to 3 days after reperfused myocardial infarction, and thus CMR, if it is to be performed, must be obtained quite early on in the admission. It is safe to perform CMR even in the first 24 hours. Yet another question is, “Why was the correlation with subsequent LV remodeling less strong than the correlation with measures on the CMR at day 2?” What one would really like to have at the time of index evaluation is the ability to predict late outcomes over and above assessment of the current state of the myocardium.

The larger question is, “In which patients should microvascular function be assessed after reperfused AMI?” The issue here is that despite exhaustive studies over the past 3 decades, little progress has been made in mitigating reperfusion injury. Intravenous adenosine had some promise in that regard in the Acute Myocardial Infarction study of Adenosine (AMISTAD‐II), in which infarct size was reduced with the higher dose and trends toward benefit were seen in larger anterior infarcts,^15^ but further studies have not been performed since that publication. A multitude of other pharmacological agents have fallen by the wayside. Does it make sense to examine the microvasculature after AMI if we have no proven therapy to improve microvascular function and thus change subsequent LV remodeling and outcome? As it stands now, all post‐AMI patients leave the hospital on 5 drugs, including aspirin, a statin, a β‐blocker, an angiotensin‐converting enzyme inhibitor, and clopidogrel—the latter if they received a stent as part of the percutaneous coronary intervention. No adjustments are made on the basis of the status of the microvasculature because these therapies are considered standard‐of‐care, and hospitals are monitored for adherence to this post‐AMI medical regimen. Until such time as we have specific therapies for microvascular injury, it might not make sense to look for it, even though we have accurate ways to assess it with CMR and now IMR. Although the physiology is certainly of great interest, no method of assessing microvascular function after reperfused AMI has reached the current high bar of practicality and clinical utility.
